# Quality of YouTube™ videos on the clinical use of silver fluoride

**DOI:** 10.1007/s40368-025-01102-w

**Published:** 2025-08-31

**Authors:** S. Zafar, K. YongHong Low, W. Yi Teh, S. Hong Chan, L. J. Walsh

**Affiliations:** University of Queensland, Brisbane, Australia

**Keywords:** Silver fluoride, Minimally invasive techniques, YouTube™

## Abstract

**Background:**

Silver fluoride (SF) is an effective, minimally invasive dental caries treatment, but the quality, reliability, and adherence to manufacturers’ instructions for use (IFU) in YouTube™ videos on SF usage remain uncertain.

**Aim:**

To evaluate the reliability and quality of YouTube™ videos on the clinical usage of SF and assess their adherence to their IFU.

**Design:**

A cross-sectional analysis of 78 YouTube™ videos was conducted. Reliability was assessed with modified DISCERN tool, source quality with Journal of American Medical Association (JAMA) benchmark criteria, production quality with Audio-Visual Quality tool, and content usefulness with Total Content Evaluation index. Adherence to clinical instructions was assessed with IFU Adherence Evaluation tool. Correlations between video characteristics and engagement metrics were analysed.

**Results:**

Videos had moderately high reliability (median mDISCERN score: 4 and IQR: 3–4) and usefulness (median TCE score: 5 and IQR: 4–6), but source quality was lower (median JAMA score: 2 and IQR: 2–3). Adherence to IFU varied between SF brands, with videos demonstrating at least 60% of steps. Dental professionals were the most frequent video producers, but no significant quality differences were observed between sources.

**Conclusion:**

YouTube™ offers potential as a learning resource for SF usage, although stricter guidelines are needed to ensure accurate information is provided.

**Supplementary Information:**

The online version contains supplementary material available at 10.1007/s40368-025-01102-w.

## Introduction

Dental caries remains a widespread oral health issue, affecting diverse populations across the lifespan, including paediatric, special needs, and geriatric patients ((WHO), 2022). Managing caries lesions in these vulnerable groups often presents unique challenges (Schmoeckel et al. 2020). Conventional treatments frequently involve invasive procedures, such as restorations, pulpal therapy, or extractions, and can be traumatic for patients who struggle with cooperation, such as the paediatric and special needs population (Schmoeckel et al. 2020; Gao et al. 2021). These interventions may also necessitate sedation or general anaesthesia (GA), posing additional risks and complications (Schmoeckel et al. 2020; Hu et al. 2018).

Silver fluoride (SF) refers broadly to topical agents including but not limited to silver diammine fluoride (SDF). SF has emerged as a minimally invasive and effective alternative in caries management (Hu et al. 2018). SF is a topical solution that combines the antimicrobial effects of silver with the remineralizing properties of fluoride, offering a simpler, atraumatic method for arresting carious lesions (Hu et al. 2018). Its application can significantly reduce the need for more invasive procedures, particularly in populations where traditional interventions are not ideal (Hu et al. 2018; Gao et al. 2020). Despite its clinical advantages, one of the primary concerns associated with SF is the permanent black staining it causes on arrested carious lesions, which may raise aesthetic concerns, especially on anterior teeth (Schmoeckel et al. 2020; Dhar et al. 2023).

In 2021, the World Health Organization (WHO) recognised the importance of SF in global dental care by including it on the WHO list of essential medicines, further emphasising its role in managing caries, particularly in populations with lack of access to dental services (WHO 2021). In Australia, SF is approved by the Therapeutic Goods Administration (TGA) for treating dentinal hypersensitivity (Do 2020). Its off-label use for caries lesion arrest has been recommended by the Australian Dental Association (Do 2020; Gao et al. 2021). However, the absence of formal clinical guidelines for SF application in caries lesion management reflects a significant gap in its integration into dental practice (Do 2020).

At the educational level, there is also a notable deficiency in formal training on the clinical use of SF in both undergraduate and postgraduate dental programs in Australia (Gao et al. 2021). Many dental students and practitioners increasingly turn to online platforms such as YouTube™ to supplement their learning (Dias da Silva et al. 2022). However, the unregulated nature of such content raises concerns about the accuracy and reliability of clinical information presented (Li et al. 2015). As SF gains broader use, it becomes crucial to evaluate the quality of these online resources, particularly in comparison to established clinical protocols, such as the manufacturer's instructions for use (IFU).

This study aimed to assess the reliability and quality of YouTube™ videos on the clinical usage of SF. Another aim of the study is to assess how accurate does the videos that demonstrate the clinical application of SF adhere to the manufacturer’s IFU. By evaluating the content against video assessment tools and the manufacturer’s IFU, this research seeks to identify potential gaps in the information available to practitioners and contribute to the development of formal recommendations for SF use in countries lacking specific protocols, such as Australia.

## Materials and methods

### Ethics approval

The University of Queensland Human Research Ethics Review Committee granted an exempted from ethics (Approval No: 2023/HE001838). The videos used in the study were sourced from YouTube™ and were all accessible by the general public. In addition to the strictly observational nature of the study, no patient-related data, viewer comments or user-generated content were included, thereby ensuring no breach of confidentiality or data protection.

### Initial video selection and search strategy

A cross-sectional study was conducted to assess the quality and reliability of YouTube™ videos on the clinical use of silver fluoride (SF). A search was conducted on February 16, 2024, using the search terms “silver fluoride” and “silver diamine fluoride” on the YouTube™ platform with results sorted by the default “Relevance” option. The search was conducted in incognito mode to eliminate bias from previous user history. The first 100 videos for each search term were selected for evaluation, following a methodology similar to previous studies (Huang et al. 2020). The Uniform Resource Locators (URLs) of the 200 videos were documented in a Microsoft Excel spreadsheet (Microsoft, Redmond, Seattle, WA, USA).

Videos were included if they were: (1) related to the clinical use of silver fluoride; (2) in the English language; and (3) were available for viewing on the search date. The exclusion criteria were: (1) duplicate videos; (2) irrelevant videos; (3) non-English; and (4) longer than 30 min.

Duplicates were identified by comparing video titles, URLs, and the video’s channel. Screening was performed by reviewing video titles and thumbnails after eliminating duplicates. In the case of duplicate videos, the version with the higher view count was retained. The full video content of the remaining videos was assessed against the predefined inclusion and exclusion criteria.

Videos longer than 30 min were excluded to reflect typical viewer engagement patterns and the practicality of clinical learning. It has been demonstrated that there is an inverse relation between the average viewing rate and user retention over video length (Dias da Silva et al. 2019).

After applying the inclusion and exclusion criteria, 78 videos were selected for assessment. Video characteristics, including upload date, number of subscribers, views, comments and likes, were recorded for each video. The videos were categorised by upload source as followed: (1) University or academic sources; (2) Dental professionals; (3) Professional dental organisations; (4) Commercial brands; and (5) Individual users.

### Video evaluation and assessment tools

To assess the quality and reliability of the videos, a total of five video assessment tools, including both validated tools and custom indices were used (refer to Sect. 2.3.1–2.3.5). The file containing the 78 videos were duplicated into separate files for independent assessment by investigators 1 and 2. All videos were evaluated independently by the two investigators, with discrepancies resolved through consensus with investigator 3.

### Journal of American Medical Association benchmark criteria

The Journal of American Medical Association benchmark criteria (JAMA) were originally introduced by (Silberg et al. 1997) and have been used extensively in other studies to evaluate the credibility of online information. The scoring ranges from 0 to 4, assessing authorship, attribution, disclosure, and currency of the content (Table 1).

**Table 1:** Assessment criteria of video content, quality, reliability, and production quality using the TCE score, JAMA criteria, mDISCERN tool and AVQ score respectively

Assessment of content: total content evaluation	Score
1. Is the definition of silver fluoride clear and accurate?	1
2. Does the video mention tooth selection?	1
3. Does the video mention patient selection?	1
4. Does the video mention the advantage/s of silver fluoride?	1
5. Does the video mention the disadvantage/s of silver fluoride?	1
6. Does the video demonstrate clinical usage of silver fluoride?	1
7. Does the video mention about follow ups?	1
Total score range	0–7

Assessment of quality: Journal of American Medical Association benchmark criteria	Score
Authorship: Authors and contributors, their affiliations, and relevant credentials should be provided	1
Attribution: References and sources for all content should be listed clearly, and all relevant copyright information noted	1
Disclosure: Website “ownership” should be prominently and fully disclosed, as should any sponsorship, advertising, underwriting, commercial funding arrangements or support, or potential conflicts of interest	1
Currency: Dates that content was posted and updated should be indicated	1
Total score range	0–4

Assessment of reliability: modified DISCERN tool	Score
1. Is the video clear, concise and understandable?	1
2. Are valid sources cited?	1
3. Is the information provided balanced and unbiased?	1
4. Are additional sources of information listed?	1
5. Does the video address areas of controversy/uncertainty?	1
Total score range	0–5

Assessment of production quality: Audio-Visual Quality (AVQ)	Score
3—Excellent—Clear, professional editing 2—Average—Non-professional editing 1—Poor—Blurry, out of focus, unintelligible 0—Unable to view	0–3

### Modified DISCERN tool

The DISCERN instrument is a validated tool used for assessing the reliability of online health information (Charnock et al. 1999). It has been modified by various studies for the assessment of health information presented in the video format (Charnock et al. 1999; Delli et al. 2016). The modified DISCERN (mDISCERN) tool uses five questions and the scoring ranges from 0 to 5, with one point given for every “Yes” and zero points given for every “No” (Table 1).

### Audio-visual quality

The Audio-Visual Quality (AVQ) scale was used in a previous study to evaluate the technical quality of each video, with scores ranging from 0 to 3 (Singh et al. 2018). Factors considered include clarity, professionalism in editing, and whether the video was visually and audibly intelligible (Table 1).

### Total content evaluation

A custom Total Content Evaluation (TCE) index was developed to specifically assess the depth and completeness of information on the clinical use of SF using the guidelines on SF by the American Academy of Pediatric Dentistry (AAPD) (AAPD 2022; Dentistry, 2018). The 7-point index included the following elements of the definition of SF, patient selection, tooth selection, advantages, disadvantages, clinical application, and follow-up (Table 1).

### Instruction-for-use adherence evaluation

For videos demonstrating the steps of SF clinical application, adherence to the manufacturer’s Instructions for Use (IFU) was assessed using the IFU Adherence Evaluation (IAE) scoring system. This tool was developed based on the IFU from two prominent SF brands: Advantage Arrest™ (Elevate Oral Care, West Palm Beach, FL, USA) and Riva Star™ (SDI, Bayswater, Victoria, Australia). (Care 2018; SDI 2020) Videos were scored based on how accurately the steps outlined in the manufacturer’s IFU were followed (Table 2).

**Table 2:** IAE scoring system adapted from the respective brands’ IFU

Advantage arrest™ IFU steps	Score
1. Isolate the affected area of the tooth with cotton rolls or protect the gingival tissue of the affected tooth with petroleum jelly. Alternatively, a rubber dam can be used to isolate the area	1
2. Clean and dry the affected tooth surface	1
3. For up to 5 treated sites per patient, dispense 1–2 drops of solution into a disposable dappen dish. Transfer material directly to the tooth surface with an applicator	1
4. Allow to air dry, do not rinse	1
*Advantage Arrest™ Total IAE Score Calculation* = *Score/4* × *100%*	100%

Riva Star™ IFU steps	Score
1. Using a non-fluoride prophylaxis paste in a rubber cup, clean the tooth/teeth to be treated and abutting teeth	1
2. Assess condition of teeth. Only teeth with sound dentine and enamel should be treated	1
3. Isolate area to be treated with cotton rolls	1
4. Dry teeth to be treated and adjoining gingival tissues	1
5. Apply a small amount of Gingival Barrier to the tissues, slightly overlapping the tooth (< 1 mm) and interproximal spaces. Light cure in a fanning motion. Alternatively, a rubber dam can be used for isolation	1
6. Protect any exposed gingival tissues and lips with petroleum jelly/cocoa butter taking care not to contaminate treatment site	1
7. Riva Star Step 1: Using the silver brush provided, pierce through the foil of the silver capsule and carefully apply solution to treatment site only	1
8. Riva Star Step 2: Immediately after, use green brush provided, pierce through the foil of the green capsule and apply a generous amount of the solution to the treatment site until the creamy white precipitate turns clear	1
9. Blot dry	1
10. Remove all protective/isolation materials used in the mouth	1
11. Discard used capsules and brushes in accordance with local regulations	1
*Riva Star™ Total IAE Score Calculation* = *Score/11* × *100%*	100%

To ensure consistency and comparability, only videos featuring commonly used brands, including Advantage Arrest™ and Riva Star™, were included. Videos that did not show the brand they used or used brands that were only featured in one video were excluded from analysis as their inclusion would not allow for meaningful comparisons with the other brands that were more prominent.

### Statistical data analysis

All statistical analyses were performed with the Jamovi 2.3.24 software (The Jamovi Project, Sydney, NSW, Australia). Non-normal distribution of quantitative data (video duration, total views, likes, comments, time since upload, JAMA, mDISCERN, AVQ, TCE and Advantage Arrest™ IAE score) was determined using the Shapiro–Wilk test (*P* < 0.05), with the exception of Riva Star™ IAE score, which displayed a normal distribution (*P* > 0.05).

Descriptive statistics were used to summarise data, with the continuous variables presented as mean and standard deviation (SD) and median and interquartile range (IQR) and categorical data expressed as number (*n*) and percentage (%). For group comparisons, the Kruskal–Wallis test was used to assess for differences between videos by source, while the Spearman’s correlation analysis was applied to explore associations between video characteristics and assessment scores. A *P*-value of < 0.05 was considered statistically significant.

Spearman’s rho (*ρ*) measures the strength and direction of monotonic relationships, with values interpreted as: 0.00–0.30 (negligible), 0.30–0.50 (low), 0.50–0.70 (moderate), 0.70–0.90 (high), and 0.90–1.00 (very high) (Mukaka 2012).

The Cohen’s kappa coefficient was calculated to evaluate the degree of interrater agreement between the two investigators of the video assessment scores, with kappa (*κ*) values interpreted as follows: < 0.20 (slight), 0.21–0.40 (fair), 0.41–0.60 (moderate), 0.61–0.80 (substantial), and > 0.81 (almost perfect agreement) (McHugh 2012).

## Results

**Fig. 1:**
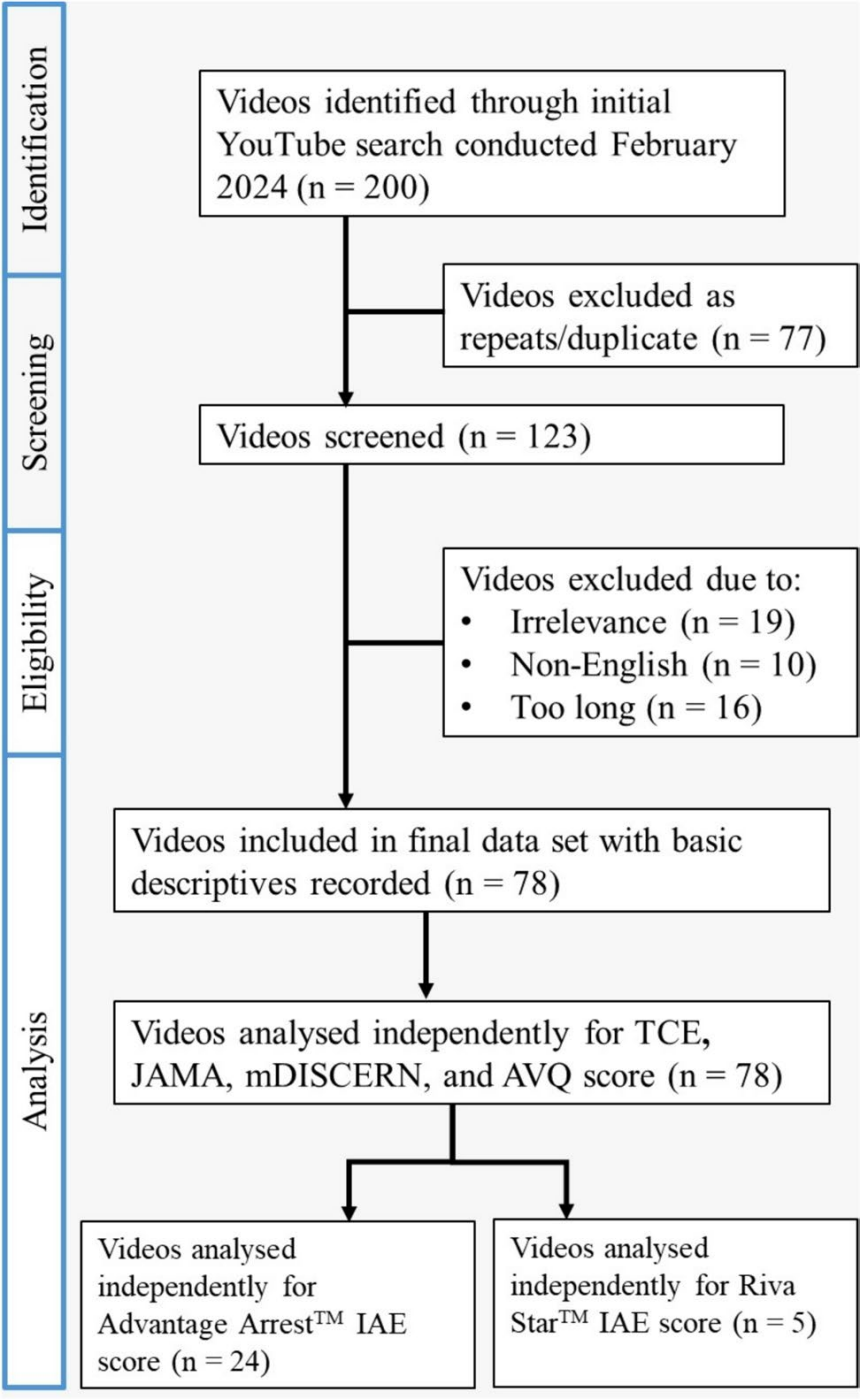
Flowchart demonstrating video selection process

A total of 200 videos were screened for each of the two search terms (“silver fluoride” and “silver diamine fluoride”). The predefined eligibility criteria excluded 122 videos: 77 were duplicates, 19 were irrelevant, 16 videos were longer than 30 min and 10 videos were not in the English language. A total of 78 videos were included for the analysis, with 29 videos further selected for analysis using the IAE scoring system, 24 of which used the Advantage Arrest™ brand and 5 of which used the Riva Star™ brand (Supplemental data—Tables 1 and 2). A flowchart of the video screening and selection process is given in Fig. 1.

Table 3 summarises the video characteristics, assessment scores and video source. Most videos had a short mean duration of 5.36 ± 5.51 min, and moderate engagement metrics. Video assessment scores indicated moderate content usefulness, low source quality, high reliability, and average production quality. The majority of videos were produced by dental professionals, followed by professional organisations and commercial brand sources. Patient selection was the most consistently addressed topic, while follow-up guidance was least frequently mentioned.

**Table 3:** Descriptives of video characteristics and video assessment scores, and frequency of videos by source and by TCE score

Video characteristics	Videos (*n*)	Mean ± SD	Median (IQR)
Duration (min)	78	5.36 ± 5.51	2.8 (1.9, 7.8)
Number of views (*n*)	78	15,543.54 ± 55,304.11	1863 (422, 10,795)
Number of likes (*n*)	78	78.99 ± 195.67	22 (2, 81)
Number of comments (*n*)	78	8.01 ± 24.33	1 (0, 5)
Number of subscriptions (*n*)	78	62,550.29 ± 433,918.44	1275 (282, 18,050)
Time since upload (day)	78	1607.12 ± 812.44	1498 (1144, 2177)
TCE score	78	5.01 ± 1.24	5 (4, 6)
JAMA score	78	2.31 ± 0.54	2 (2, 3)
mDISCERN score	78	3.38 ± 1.15	4 (3, 4)
AVQ score	78	2.49 ± 0.50	2 (2, 3)
Advantage Arrest IAE score	24	83.3 ± 25.2	100.0 (75.0, 100.0)
Riva Star IAE score	5	69.1 ± 15.2	72.7 (63.6, 81.8)
Video source	*n* (%)		
University	1 (1.3)		
Professional organisations	11 (14.1)		
Dental professionals	57 (73.1)		
Commercial brands	4 (5.1)		
TCE score	Yes (*n* [%])	No (*n* [%])	
Definition of SF	51 (65.4)	27 (34.6)	
Tooth selection	69 (88.5)	9 (11.5)	
Patient selection	77 (98.7)	1 (1.3)	
Advantages of SF	62 (79.5)	16 (20.5)	
Disadvantages of SF	57 (73.1)	21 (26.9)	
Clinical application of SF	45 (57.7)	33 (42.3)	
Follow up	30 (38.5)	48 (61.5)	

The Kruskal–Wallis test showed a significant difference in video duration by source, with professional organisations producing the longest videos, while dental professionals and individual users had notably shorter videos (Supplemental data Table 1). There were no other statistically significant differences in other characteristics or assessment scores across sources.

Spearman’s correlation analysis showed significant positive correlations between TCE and mDISCERN scores, and between TCE and AVQ scores, indicating that more comprehensive content tends to be more reliable and better produced (Table 4).

**Table 4:** Spearman’s correlation test for video characteristics and video assessment tools

		TCE	JAMA	mDISCERN	AVQ
Duration	*r*	**0.379**	0.084	**0.237**	− 0.134
	*p*	< 0.001*	0.463	0.037*	0.241
View	*r*	0.110	**0.330**	0.205	0.077
	*p*	0.337	0.003*	0.072	0.500
Likes	*r*	0.222	**0.354**	**0.294**	0.115
	*p*	0.051	0.001*	0.009*	0.317
Comments	*r*	0.141	0.114	0.188	− 0.070
	*p*	0.218	0.320	0.098	0.544
Subscribers	*r*	0.200	**0.342**	**0.413**	0.185
	*p*	0.080	0.002*	< 0.001*	0.104
Time since upload	*r*	− 0.103	0.165	− 0.184	− **0.231**
	*p*	0.368	0.150	0.106	0.042*
TCE	*r*	–	–	–	–
	*p*	–	–	–	–
JAMA	*r*	0.051	–	–	–
	*p*	0.656	–	–	–
mDISCERN	*r*	**0.621**	**0.364**	–	–
	*p*	< 0.001*	0.001*	–	–
AVQ	*r*	**0.372**	− 0.030	0.167	–
	*p*	< 0.001*	0.792	0.143	–

Bolded values in the table indicate statistical significance at *P* value 0.05

*r* Spearman’s rho

^*^P < *0.05*

TCE scores and mDISCERN scores were both weakly positively correlated with video duration (*r* = 0.379, *P* < 0.001 and *r* = 0.237, *P* = 0.037 respectively)), implying that longer videos tend to be more comprehensive and reliable. mDISCERN scores also showed weak positive correlations with engagement metrics such as likes (*r* = 0.294, *P* = 0.009) and subscribers (*r* = 0.418, *P* < 0.001)., suggesting that videos with more reliability attract more interaction.

Similarly, JAMA scores also showed weak positive correlations with engagement metrics such as views (*r* = 0.330, *P* = 0.003), likes (*r* = 0.354, *P* = 0.001), and subscribers (*r* = 0.342, *P* = 0.002), also suggesting that videos with more quality attract more interaction. Additionally, there was a weak negative correlation between AVQ scores and time since upload (*r* =  − 0.231, *P* = 0.042), indicating that older videos tend to exhibit lower production quality.

**Fig. 2:**
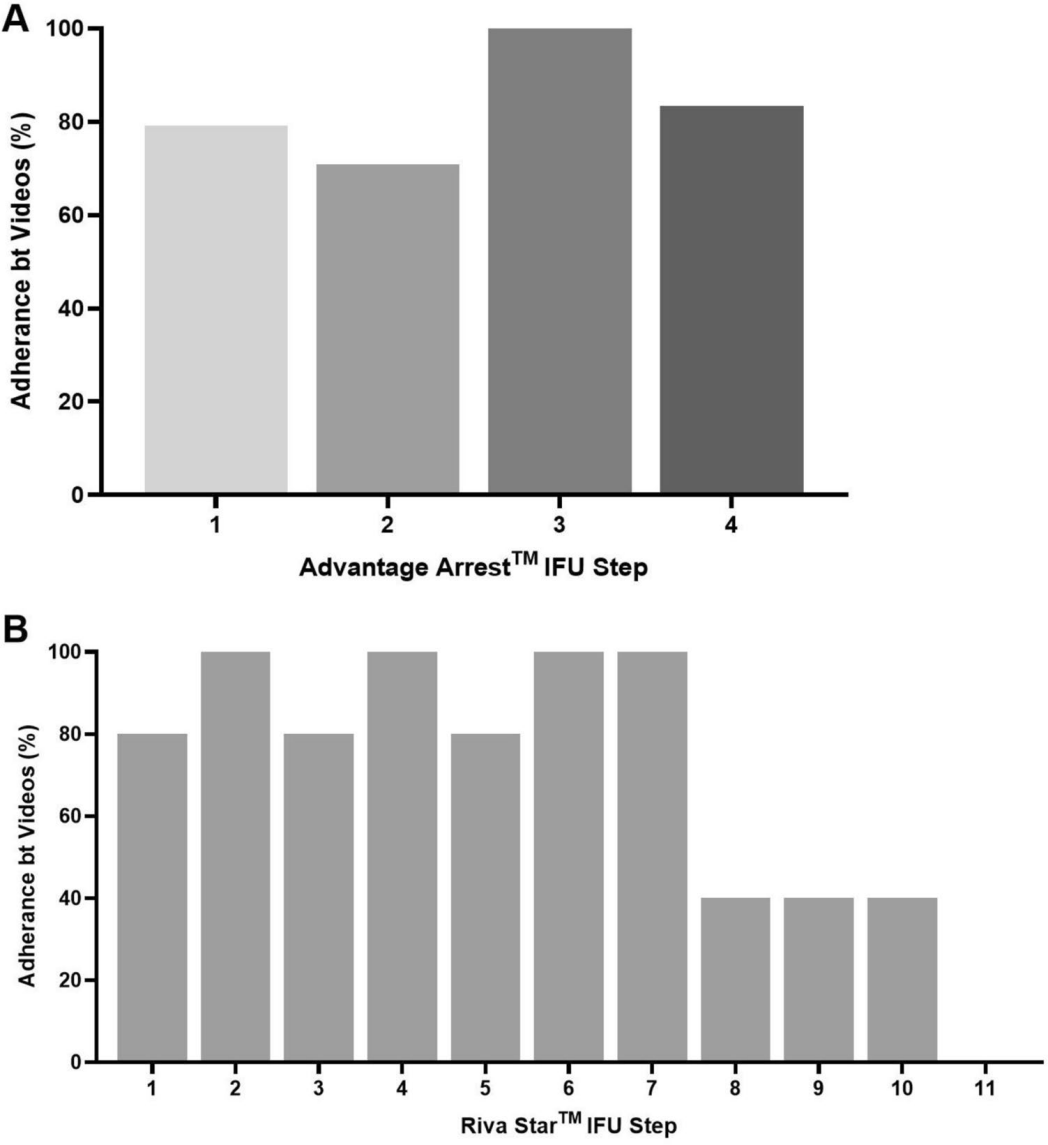
Percentage of video adherence of each step. **A** Advantage Arrest™ IFU by the 24 videos. **B** Riva Star™ IFU by the five videos

The median IAE score for Advantage Arrest™ was 100.0% (IQ*R* = 75.0–100.0), while the mean IAE score for Riva Star™ was 69.1% (± 15.2) indicating that videos on Advantage Arrest™ adhered more closely to manufacturer’s IFU compared to those demonstrating the use of Riva Star™ (Table 3). Adherence to each step of the IFU varied between videos. Figures 1 and 2 show the adherence rates for the Advantage Arrest™ and Riva Star™ brands respectively, with certain steps more consistently followed than others.

Cohen’s kappa coefficient values for interrater reliability were substantial to near perfect: TCE (*κ* = 0.672), JAMA (*κ* = 0.801), mDISCERN (*κ* = 0.672), AVQ (*κ* = 0.845), IAE for Advantage Arrest™ (*κ* = 0.619), and IAE for Riva Star™ (*κ* = 0.870), reflecting strong agreement among the two investigators.

## Discussion

This study aimed to assess the quality, reliability, and comprehensiveness of YouTube™ videos concerning SF. A total of 78 videos met the inclusion criteria, with most (73.1%) produced by dental professionals. This reflects the growing use of YouTube™ as a platform for professional education within dentistry, though variability in video quality and content were evident.

According to mDISCERN scores, content reliability was generally moderate, with significant correlations to comprehensiveness and credibility. However, variability in quality persists, highlighting the need for better quality control and evidence-based guidelines.

According to the JAMA scores, the videos demonstrated an overall low—moderate source quality, with less compliance in disclosure and attribution. Higher JAMA scores were weakly positively correlated with usefulness and popularity. This highlights the need for more consistent application of these standards in healthcare videos.

The content analysis using TCE tool revealed that a large portion of the videos was of moderately-high usefulness. However, there still exists large portion of videos that lacked crucial information which could mislead dental professionals, such as missing information on follow up.

A strength of this study is the use of validated assessment tools such as the mDISCERN, JAMA, and AVQ scoring systems, which allowed for a structured and comprehensive analysis of video quality (Cesur Aydin and Gunec 2020; Singh et al. 2018; Er and Gülfeşan Çanakçı, 2022). Based on the present findings from the assessment tools used, the videos available on YouTube™ on SF displayed low-moderate source quality, however had moderately-high reliability, usefulness and production quality. Furthermore, videos that demonstrated the clinical steps of using SF generally had quite high accuracy when compared to the brand’s IFU.

Spearman’s analysis revealed a moderate correlation between TCE and mDISCERN scores, indicating that better content reliability correlates with video usefulness. The weak positive correlation between JAMA and mDISCERN scores suggests videos with higher video source quality are also more reliable. These findings suggest that content reliability with higher video source quality videos are more useful for clinical learning and may help improve evidence-based practice. Production quality showed a weak but significant positive correlation with video usefulness, suggesting its importance in enhancing the learning experience.

The majority of videos (98.7%) addressed patient selection in the clinical usage of SF, as assessed by the TCE criteria. However, most focused on the paediatric application of SF, with very few addressing its use in geriatric populations or individuals with special needs. Given that SF is minimally invasive, cost-effective, and simple to use, its application in vulnerable populations such as the elderly and special needs patients should be emphasised more (Gao et al. 2021). The underrepresentation of these groups may reflect a gap in awareness or emphasis on broader clinical use cases for SF.

Almost 90% of the videos discussed the topic of tooth selection. Most videos focused on the off-label use of SF for carious teeth, with few addressing its effectiveness for dentinal hypersensitivity, despite studies showing SF can reduce dentinal hypersensitivity by 23–56% after a single application (Do 2020; Chan et al. 2024).

Although not a primary objective of the present study, it was observed that contraindications for clinical SF application were rarely mentioned in the videos. In particular, few videos addressed its use on teeth with clinical signs of pulpal inflammation, a contraindication highlighted in the AAPD chairside guidelines (AAPD 2022). However, recent literature suggests indirect application may be safe for teeth with reversible pulpitis, even in cases where the remaining dentine thickness was 0.25–0.5 mm. (Yan et al. 2022; Zaeneldin et al. 2022). Studies show minimal inflammatory response and caries lesion arrest in these cases (Zaeneldin et al. 2022; Yan et al. 2022). However, it is important to note that direct SF application to vital pulp remains contraindicated due to its potential for severe pulpal inflammation and necrosis (Zaeneldin et al. 2022). As a result, given the conflicting information between sources, this further highlights the need for standardised protocols regarding clinical SF usage to ensure appropriate application of this valuable therapeutic agent.

Only around 40% of the videos discussed the importance of follow-up. Research suggests that a single application of SF has effectiveness that can range from 47 to 90% depending on lesion size and location of the lesion (Fung et al. 2018). Without proper follow-up, there is a risk of continued caries progression (AAPD 2022; Fung et al. 2018).

A significant disparity was observed in the adherence of videos to the manufacturer’s IFU. Videos featuring Advantage Arrest™ consistently outperformed those featuring Riva Star™ in following the IFU steps. However, despite this finding, the Riva Star™ videos were potentially more reliable than what the results show, which could be explained through several reasons.

Firstly, for the videos that used Riva Star™, a common step omitted was step 8—potassium iodide (KI) application. Interestingly, two studies have shown that the addition of KI to SF reduces its caries-arresting effect due to the formation of silver iodide instead (Sorkhdini et al. 2020; Alvarez-Marín et al. 2024). Furthermore, the discolouration caused by SF was not prevented in the long term from use of KI, even though it significantly reduced the discolouration caused by SF immediately post-operatively (Aly and Yousry 2022; Roberts et al. 2020).

Secondly, only 40% of the videos demonstrated step 10—removal of isolation materials, whereas none of the videos demonstrated step 11—proper disposal of materials. This could have resulted in the IAE score for Riva Star™ videos to be much lower than they would be if the Advantage Arrest™ and Riva Star™ IFUs were more similar in their IFU steps. These steps are not critical to the clinical application of SF and outcome and could have been omitted.

Our findings align with previous research showing YouTube™ can be a valuable resource, though users must critically assess content (Yan et al. 2022). Similar to other studies, we found universities contribute only a small portion of educational material on YouTube™ (Dias da Silva et al. 2019, 2022). Additionally, unlike another study on dental radiology, which found university-produced videos were of higher quality, the present results suggest non-university sources can produce content of comparable value on SF, likely due to the straightforward nature of SF clinical application (Cesur Aydin and Gunec 2020). Contrary to previous studies that broadly deem YouTube™ to be an unreliable platform for dental education, the present results indicate that the reliability of content may vary depending on the topic, highlighting the importance of subject matter in determining video quality (Dias da Silva et al. 2019).

The dynamic nature of YouTube™ means video content can quickly become outdated, limiting the longevity of these findings, as they only represent a snapshot analysis of the videos that existed on the search date. Additionally, the exclusion of non-English videos may affect generalisability. Furthermore, conducting the search in incognito mode does not fully eliminate bias, as the search results can still be influenced by factors such as trending content or regional differences, introducing potential bias in the selection of videos retrieved during the time of the search. Finally, the small sample size, particularly from certain sources, limited the statistical power to detect group differences and thus, due to insufficient statistical power, regression analyses with TCE, JAMA, mDISCERN, and AVQ as outcomes were considered but not conducted.

Using the results from the present study, future studies should explore how these videos impact the dental practitioners directly, and whether they improve their knowledge and clinical usage of SF. Future studies should also consider longitudinal designs to track changes in video quality and reliability as clinical guidelines on SF get updated. Additionally, further research is needed on the role of universities and professional organisations in producing reliable, accessible content on SF for dental education.

The frequent omission of critical steps in SF application highlights the need for clinicians to critically evaluate online resources. YouTube™ can be a useful supplement but should not replace evidence-based guidelines or manufacturer’s IFU, which are reliable sources that clinicians can use to cross-reference and assess the reliability of an online clinical video.

To address gaps in dental education regarding the clinical use of SF, educators could curate and incorporate high-scoring YouTube™ videos identified using validated assessment tools from the present study into their education programs (Gao et al. 2021). Universities utilising online video content may benefit from adopting a standardised video quality checklist based on these tools used in the study, helping ensure consistency and reliability across the digital learning materials. In addition, the lack of videos addressing SF use in geriatric and special needs populations indicates a gap in education that should be addressed.

## Conclusion

This study found that the overall quality of YouTube™ videos on SF is moderate, with some videos providing valuable, accurate information. However, significant gaps remain in terms of completeness and reliability, particularly regarding patient follow-up. As YouTube™ continues to grow as an educational platform, dental professionals should be aware of its limitations and critically evaluate the content for SF clinical usage. Further engagement by professional organisations and universities could improve the quality and reliability of educational materials available online, particularly for newer treatments like SF.

## Supplementary Information

Below is the link to the electronic supplementary material.

Supplementary file1 (DOCX 47 KB)

## Data Availability

No datasets were generated or analysed during the current study.
