# Supplementary material for: Quality of YouTube™ videos on the clinical use of silver fluoride
Source: Eur Arch Paediatr Dent. 2025 Aug 31;26(6):1211–20. doi: 10.1007/s40368-025-01102-w (PMC12638426; doi:10.1007/s40368-025-01102-w)
Supplement: Supplementary file 1 — Supplementary file1 (DOCX 47 KB) [file 40368_2025_1102_MOESM1_ESM.docx]

**Supplemental Data**

**Table 5 –** Descriptives statistics stratified by source.

| Variable | University | Professional organisations | Dental professionals | Commercial brands | Individual users |  |
| --- | --- | --- | --- | --- | --- | --- |
|  | Median** | Median (IQR) | Median (IQR) | Median (IQR) | Median (IQR) | *p-*value |
| Duration (min) | 7.2 | 11.7 (6.3-13.2) | 2.5 (1.4-4.0) | 9.4 (4.2-16.5) | 2.3 (2.2-3.1) | **<0.001*** |
| View (n) | 17273 | 420 (114- 10576) | 2235 (611-10797) | 3910 (803-11537) | 855 (58-5127) | 0.523 |
| Like (n) | 157 | 2 (1-76) | 24 (3-72) | 37 (5-86) | 4 (0-35) | 0.399 |
| Comment (n) | 8 | 0 (0-1) | 1 (0-6) | 7 (1-19) | 0 (0-2) | 0.104 |
| Subscription (n) | 19800 | 469 (367-1030) | 1520 (271-15500) | 1292 (893-9368) | 26000 (4880-50300) | 0.363 |
| Time since upload (day) | 1044 | 1498 (1365-1498) | 1563 (1028-2303) | 2067 (1429-2517) | 1344 (1283-2079) | 0.759 |
| TCE | 7 | 5 (4-5) | 5 (4-6) | 6 (5.5-6.25) | 5 (4-6) | 0.417 |
| JAMA | 2 | 2 (2-3) | 2 (2-3) | 2.5 (2-3) | 2 (2-2) | 0.536 |
| mDISCERN | 4 | 3 (2-4) | 4 (3-4) | 4 (3.5-4) | 3 (2-4) | 0.821 |
| AVQ | 3 | 2 (2-2.5) | 2 (2-3) | 2.5 (2-3) | 3 (3-3) | 0.082 |

Values are presented as median (IQR)

^a^ Kruskal-Wallis test was used

**p < 0.05*

***University only has 1 video, hence IQR not represented on table.*

**Table 6**- Characteristics and sources of included videos

| **Video No.** | **Video Title** | **Video URL** | **SubscribersNo.** | **View No** | **Likes** | **Date Uploaded** | **Duration** |
| --- | --- | --- | --- | --- | --- | --- | --- |
| 1 | Silver Diamine Fluoride Placement | <https://www.youtube.com/watch?v=fPZ8XHbF-DM> | 91800 | 38301 | 269 | 24/03/2018 | 1 min 28 sec |
| 2 | Is silver diamine fluoride working? | <https://www.youtube.com/watch?v=Cz1GwfD70hA> | 15500 | 2572 | 34 | 30/11/2022 | 5 min 13 sec |
| 3 | Silver Diamine Fluoride (SDF) Application: Evidence-Based Recommendations | <https://www.youtube.com/watch?v=a0HH7GifdM4> | 60500 | 154388 | 966 | 14/11/2018 | 5 min 36 sec |
| 4 | How to apply Silver Diamine Fluoride | <https://www.youtube.com/watch?v=tELmH9jRvv8> | 1030 | 20625 | 86 | 24/06/2020 | 3 min 50 sec |
| 5 | Silver Diamine Fluoride \| Step By Step Demonstration | <https://www.youtube.com/watch?v=FdY_KEILn2I> | 73900 | 19027 | 166 | 26/12/2020 | 2 min 37 sec |
| 6 | Application of Silver Diamine Fluoride 38% | <https://www.youtube.com/watch?v=p9Tazwitcao> | 9300 | 29899 | 146 | 06/04/2018 | 3 min 29 sec |
| 7 | SDF Application Tutorial with Advantage Arrest Silver Diamine Fluoride 38% | <https://www.youtube.com/watch?v=zxlvbhUx3QE> | 9300 | 463462 | 1400 | 18/04/2017 | 3 min 40 sec |
| 8 | What You Should Know About Silver Diamine Fluoride. | <https://www.youtube.com/watch?v=bwAkXt0cCMI> | 3 | 680 | 2 | 14/06/2020 | 2 min 14 sec |
| 9 | New SDF Gel, Advantage Arrest 38% Silver Diamine Fluoride | <https://www.youtube.com/watch?v=pS-6pX_T0XY> | 9300 | 2619 | 27 | 24/02/2023 | 1 min 24 sec |
| 10 | Silver Diamine Fluoride | <https://www.youtube.com/watch?v=doan4py_m5g> | 91800 | 5693 | 72 | 21/05/2021 | 51 sec |
| 11 | How is silver diamine fluoride transforming dental caries programmes? | <https://www.youtube.com/watch?v=yVVYkHpLJC0> | 26000 | 5127 | 35 | 12/08/2020 | 3 min 3 sec |
| 12 | Silver Diamine Fluoride | <https://www.youtube.com/watch?v=_zpzWq5oTws> | 19800 | 17273 | 157 | 08/04/2021 | 7 min 12 sec |
| 13 | Silver Diamine Fluoride Application Demo - Close Up | <https://www.youtube.com/watch?v=pPZT033Kk2Y> | 282 | 49769 | 147 | 26/06/2016 | 2 min 48 sec |
| 14 | Is Silver Diamine Fluoride Replacing Sealants? | <https://www.youtube.com/watch?v=4f0DWemMYNM> | 91800 | 1179 | 23 | 16/05/2023 | 1 min 16 sec |
| 15 | Superfloss Technique Tutorial using Silver Diamine Fluoride SDF by Dr. Jeanette MacLean,DDS | <https://www.youtube.com/watch?v=nPyYpZYfrHQ> | 9300 | 38568 | 178 | 17/08/2017 | 2 min 39 sec |
| 16 | Silver Diamine Fluoride | <https://www.youtube.com/watch?v=gfV29rI9L3A> | 415 | 9457 | 35 | 13/11/2019 | 1 min 22 sec |
| 17 | Baby Cavity Treatment with Silver Fluoride? | <https://www.youtube.com/watch?v=AsfLMoonU-Q> | 18900 | 617 | 7 | 18/01/2023 | 1 min 45 sec |
| 18 | Silver Diamine Fluoride for Children and Young People | <https://www.youtube.com/watch?v=njfhmN55HWs> | 1030 | 15802 | 90 | 24/06/2020 | 6 min 42 sec |
| 19 | Everything You Need to Know About Silver Diamine Fluoride | <https://www.youtube.com/watch?v=F6gwK57Vlx4> | 32400 | 6897 | 67 | 04/09/2019 | 13 min 32 sec |
| 20 | What If Your Child Has a Cavity? Prevent and Treat! | <https://www.youtube.com/watch?v=7g_Hni42Ydg> | 29800 | 17702 | 148 | 27/01/2021 | 8 min 49 sec |
| 21 | Application of Silver Diamine Fluoride (SDF) without Aerosols | <https://www.youtube.com/watch?v=7aiyiRnXhQE> | 9300 | 28292 | 236 | 11/05/2020 | 4 min 20 sec |
| 22 | Silver Diamine Fluoride Info and Application | <https://www.youtube.com/watch?v=y5K5fdcCgdc> | 29800 | 874 | 21 | 24/04/2021 | 9 min 3 sec |
| 23 | New Way to Stop Tooth Decay: Silver Diamine Fluoride | <https://www.youtube.com/watch?v=5gjrMoQEShI> | 1050 | 10289 | 146 | 13/03/2018 | 3 min 3 sec |
| 24 | SILVER DIAMINE FLUORIDE for Incipient Caries & CHANNEL UPDATES | <https://www.youtube.com/watch?v=XMf06iWxCN0> | 44400 | 4932 | 85 | 17/10/2018 | 9 min 50 sec |
| 25 | Silver Diamine Fluoride Application | <https://www.youtube.com/watch?v=a-IgQdqM314> | 282 | 10797 | 25 | 19/11/2016 | 2 min 48 sec |
| 26 | Riva Star ( silver fluoride potassium iodide) | <https://www.youtube.com/watch?v=D_KEOiMTzxk> | 1600 | 45332 | 129 | 02/11/2010 | 2 min 26 sec |
| 27 | SMART Filling with Advantage Arrest SDF Silver Diamine Fluoride and GIC | <https://www.youtube.com/watch?v=0kiqG0z66qs> | 9300 | 29933 | 111 | 19/01/2017 | 2 min 52 sec |
| 28 | Silver Diamine Fluoride (SDF) Treatment for Cavities | <https://www.youtube.com/watch?v=KR7cJuD1npw> | 159 | 939 | 5 | 27/05/2020 | 2 min 20 sec |
| 29 | What is SDF, Silver Diamine Fluoride? Answered by Dr. Alvarez of Smile Squad LV | <https://www.youtube.com/watch?v=C7Mfo96jE4o> | 269 | 426 | 1 | 30/12/2020 | 1 min 17 sec |
| 30 | TEACHING HUNT \| CONTESTANT NO: 40 \| Silver Diamine Fluoride (SDF) \| Dr.Sadaram V S Krishna Priya | <https://www.youtube.com/watch?v=07ErtnL7E04> | 20000 | 346 | 10 | 31/10/2020 | 10 min 57 sec |
| 31 | WFPHA \| Dr. Geoff Knight on Silver Diamine Fluoride in Australia | <https://www.youtube.com/watch?v=_U0XXLRmX3E> | 267 | 734 | 7 | 10/01/2020 | 13 min 5 sec |
| 32 | Silver Diamine Fluoride Informed Consent | <https://www.youtube.com/watch?v=qRLFup2QJG0> | 5520 | 2235 | 0 | 01/09/2017 | 1 min 11 sec |
| 33 | WFPHA \| Q&A on Silver Diamine Fluoride in Transforming Community Dental Caries Programs | <https://www.youtube.com/watch?v=AUvgGIe7RBA> | 567 | 420 | 0 | 10/01/2020 | 13 min 17 sec |
| 34 | ORAL SCIENCE \| Advantage Arrest : Silver Diamine Fluoride - Launch at the PDC (Vancouver) | <https://www.youtube.com/watch?v=1rUCxC4mYIo> | 891 | 922 | 3 | 05/04/2017 | 54 sec |
| 35 | WFPHA \| Dr. Richard Niederman on Silver Diamine Fluoride in the USA | <https://www.youtube.com/watch?v=VO_zus2FdLA> | 467 | 116 | 0 | 10/01/2020 | 11 min 39 sec |
| 36 | POHIC16 Silver Fluoride & Potassium Iodine | <https://www.youtube.com/watch?v=u1zTA4s09_w> | 752 | 611 | 3 | 02/06/2016 | 10 min 12 sec |
| 37 | Cavity-Fighting SDF Treatment for Toddlers \| Vanilla Smiles \| Best Dental Clinic in Pune | <https://www.youtube.com/watch?v=s5EoTzNxDYM> | 766 | 15154 | 65 | 25/06/2019 | 1 min 20 sec |
| 38 | TIPisode Silver Diamine Fluoride: A Staple Product For Your Op | <https://www.youtube.com/watch?v=Na6rSO422Jo> | 1500 | 20 | 1 | 10/09/2021 | 8 min 21 sec |
| 39 | SMART Technique: caries stabilization | <https://www.youtube.com/watch?v=grZ0PsyG7Ck> | 6010 | 41435 | 277 | 14/02/2019 | 4 min |
| 40 | Professor Edward Lynch discusses the clinical use of Silver Diamine Fluoride (SDF) | <https://www.youtube.com/watch?v=ANBaB6_wS2I> | 1690 | 444 | 6 | 02/11/2021 | 25 min 21 sec |
| 41 | Silver Diamine Fluoride for Cavity Prevention in Kids \| Vanilla Smiles \| Best Dental Clinic in Pune | <https://www.youtube.com/watch?v=mjVBQ46v0mQ> | 766 | 692 | 3 | 07/02/2019 | 1 min 19 sec |
| 42 | Alternative to Cavities- Silver Diamine Fluoride- Palos Pediatric Dentistry | <https://www.youtube.com/watch?v=f2wjj7yTBc0> | 22 | 974 | 8 | 07/07/2016 | 1 min 28 sec |
| 43 | WFPHA \| Take-Home Messages on Silver Diamine Fluoride - Transforming Dental Caries Programmes | <https://www.youtube.com/watch?v=yAuNFcLxh1Q> | 467 | 112 | 1 | 10/01/2020 | 19 min 51 sec |
| 44 | Silver Diamine Fluoride Application Demo | <https://www.youtube.com/watch?v=AUmDgCWMpMk> | 282 | 7258 | 25 | 26/06/2016 | 2 min 49 sec |
| 45 | How I Use Silver Diamine Fluoride in my Mobile Practice | <https://www.youtube.com/watch?v=uiKAPASx0x8> | 29800 | 222 | 5 | 30/06/2019 | 9 min 46 sec |
| 46 | Benefits of SDF (Silver Diamine Fluoride) & Placement Demonstration | <https://www.youtube.com/watch?v=6_PFur3hcBY> | 4 | 316 | 3 | 01/09/2020 | 1 min 58 sec |
| 47 | Silver Diamine Fluoride done by Nunavut Oral Health Project | <https://www.youtube.com/watch?v=R3QPbGNPPCY> | 0 | 52 | 0 | 12/06/2020 | 1 min 57 sec |
| 48 | Silver Diamine Fluoride \| SDF \| Lunch with Expert - Dr. Stewart Ray | <https://www.youtube.com/watch?v=6aVaf8JQiXI> | 824 | 535 | 16 | 09/02/2019 | 18 min 4 sec |
| 49 | Silver Diamine Fluoride | <https://www.youtube.com/watch?v=U43m3bBtCvA> | 64 | 774 | 0 | 26/02/2018 | 3 min |
| 50 | SDF (Silver Diamine Fluoride) Dr Bell Tip #1 | <https://www.youtube.com/watch?v=3RPH8KFucdo> | 20 | 245 | 0 | 14/11/2018 | 33 sec |
| 51 | Silver Diamine Fluoride II Pediatric Dentistry | <https://www.youtube.com/watch?v=yqCdVOeiYy0> | 271 | 100 | 9 | 19/01/2021 | 2 min 29 sec |
| 52 | What is Silver Diamine Fluoride and is it a good idea for my child? | <https://www.youtube.com/watch?v=ymXk0EZ9z1I> | 15 | 28 | 0 | 13/11/2020 | 2 min 33 sec |
| 53 | Silver Diamine Fluoride | <https://www.youtube.com/watch?v=7f2WvnWyGtE> | 2 | 1 | 0 | 18/10/2023 | 2 min 9 sec |
| 54 | Silver Diamine Fluoride at RiverWest Dental | <https://www.youtube.com/watch?v=6fazJXe9_ok> | 17 | 66 | 0 | 08/02/2022 | 2 min 24 sec |
| 55 | Silver Diamine Fluoride Discussion | <https://www.youtube.com/watch?v=KjjiLPEfByM> | 282 | 2598 | 15 | 26/06/2016 | 3 min 45 sec |
| 56 | Your Healthy Family: The pros and cons of silver diamine flouride | <https://www.youtube.com/watch?v=YpUoQfjInog> | 50300 | 855 | 4 | 08/06/2018 | 2 min 9 sec |
| 57 | Silver Diamine Fluoride | <https://www.youtube.com/watch?v=fgriphJgizY> | 21 | 22 | 0 | 12/01/2022 | 47 sec |
| 58 | Basic Silver Diamine Fluoride SDF Application for Occlusal Caries | <https://www.youtube.com/watch?v=F0gNYa763KE> | 9300 | 10417 | 29 | 23/09/2017 | 2 min 30 sec |
| 59 | PEDIATRIC DENTISTRY \| SILVER DIAMINE FLUORIDE \| SDF | <https://www.youtube.com/watch?v=MXxqFXjUflg> | 2840 | 2878 | 67 | 25/01/2023 | 12 min 28 sec |
| 60 | Silver Diamine Fluoride #shorts | <https://www.youtube.com/watch?v=4wYAIAH_DTg> | 3520 | 2405 | 59 | 09/11/2021 | 19 sec |
| 61 | YUCK I Put Silver Diamine Fluoride On MY Teeth! | <https://www.youtube.com/watch?v=wiKUZaxzrFQ> | 29800 | 779 | 11 | 06/11/2019 | 5 min 24 sec |
| 62 | Superfloss SDF Tutorial for interproximal cavities using Advantage Arrest Silver Diamine Fluoride 38 | <https://www.youtube.com/watch?v=Q8dgVQG8i0o> | 9300 | 17968 | 64 | 18/04/2017 | 2 min 12 sec |
| 63 | Silver Diamine Fluoride (SDF) Application | <https://www.youtube.com/watch?v=-iLJLaeA9LA> | 282 | 1719 | 11 | 06/04/2018 | 2 min 24 sec |
| 64 | Children and Laura the dentist explain all about Silver Diamine Fluoride. | <https://www.youtube.com/watch?v=qHz8CT_rNMM> | 1030 | 5350 | 65 | 15/12/2020 | 5 min 52 sec |
| 65 | Advantage Arrest : Silver Diamine Fluoride | <https://www.youtube.com/watch?v=lUlu7PC7EDM> | 894 | 25457 | 142 | 28/02/2017 | 5 min 19 sec |
| 66 | Silver Diamine Fluoride Case Study | <https://www.youtube.com/watch?v=ayw5TwoKWFU> | 91800 | 1110 | 26 | 01/09/2023 | 1 min 7 sec |
| 67 | Silver diamine fluoride (sdf) application demo | <https://www.youtube.com/watch?v=EFVkqtVRlKs> | 1520 | 2566 | 24 | 20/11/2017 | 37 sec |
| 68 | An overview of silver diamine fluoride | <https://www.youtube.com/watch?v=xIPtOMwzr7s> | 44500 | 15971 | 205 | 23/08/2017 | 23 min 41 sec |
| 69 | SDF - Things to know about Silver Diamine Fluoride | <https://www.youtube.com/watch?v=UXe1QLF8oew> | 27100 | 2007 | 38 | 17/07/2020 | 6 min 31 sec |
| 70 | Root Canal Alternative and Prevention: Silver Diamine Fluoride Treatment | <https://www.youtube.com/watch?v=ZwLzU7cXmTU> | 129 | 261 | 0 | 04/09/2022 | 31 sec |
| 71 | WFPHA \| Dr. Prathip Phantumvanit on Silver Diamine Fluoride in Thailand | <https://www.youtube.com/watch?v=2HA_BKVd_Go> | 469 | 117 | 1 | 10/01/2020 | 12 min 15 sec |
| 72 | SDF application tutorial. Non aerosol producing dentistry! | <https://www.youtube.com/watch?v=y4Zi_mzwDJw> | 1520 | 2893 | 39 | 26/05/2020 | 32 sec |
| 73 | DFCsmiles - Silver Diamine Fluoride, SDF procedure | <https://www.youtube.com/watch?v=Ty1OcJ_ZAv4> | 6 | 806 | 5 | 28/10/2017 | 1 min 52 sec |
| 74 | Silver Diamine Fluoride: Game Changer in Dental Public Health | <https://www.youtube.com/watch?v=M9nBs0IK1EI> | 4880 | 58 | 0 | 19/12/2020 | 2 min 16 sec |
| 75 | GCDFund: Covid-19 and the role of Silver Diamine Fluoride in emergency dental care (Part 1) | <https://www.youtube.com/watch?v=YYUwLH0cgQI> | 63 | 51 | 2 | 20/04/2020 | 18 min 22 sec |
| 76 | What is Silver Diamine Fluoride? | <https://www.youtube.com/watch?v=7kbZHb_asKU> | 0 | 13 | 0 | 07/11/2023 | 2 min 24 sec |
| 77 | This new treatment could make your dental visit more bearable | <https://www.youtube.com/watch?v=rsZgLB-8-HM> | 3840000 | 10789 | 98 | 04/01/2018 | 8 min 41 sec |
| 78 | GCDFund: Covid-19 and the role of Silver Diamine Fluoride in emergency dental care (Part 2) | <https://www.youtube.com/watch?v=r267_U8iw9M> | 63 | 23 | 0 | 20/04/2020 | 7 min 59 sec |
